# Structure of the C-terminal domain of TRADD reveals a novel fold in the death domain superfamily

**DOI:** 10.1038/s41598-017-07348-9

**Published:** 2017-08-01

**Authors:** Ning Zhang, Wensu Yuan, Jing-Song Fan, Zhi Lin

**Affiliations:** 1 School of Life Sciences, Tianjin University, Tianjin, 300072 P.R. China; 2 Department of Biological Sciences, National University of Singapore, Singapore, 117543 Singapore; 3 Department of Physiology, National University of Singapore, Singapore, 117593 Singapore; 4 Life Sciences Institute, National University of Singapore, Singapore, 117456 Singapore

## Abstract

The TNFR1-associated death domain protein (TRADD) is an intracellular adaptor protein involved in various signaling pathways, such as antiapoptosis. Its C-terminal death domain (DD) is responsible for binding other DD-containing proteins including the p75 neurotrophin receptor (p75^NTR^). Here we present a solution structure of TRADD DD derived from high-resolution NMR spectroscopy. The TRADD DD comprises two super-secondary structures, an all-helix Greek key motif and a β-hairpin motif flanked by two α helices, which make it unique among all known DD structures. The β-hairpin motif is essential for TRADD DD to fold into a functional globular domain. The highly-charged surface suggests a critical role of electrostatic interactions in TRADD DD-mediated signaling. This novel structure represents a new class within the DD superfamily and provides a structural basis for studying homotypic DD interactions. NMR titration revealed a direct weak interaction between TRADD DD and p75^NTR^ DD monomers. A binding site next to the p75^NTR^ DD homodimerization interface indicates that TRADD DD recruitment to p75^NTR^ requires separation of the p75^NTR^ DD homodimer, explaining the mechanism of NGF-dependent activation of p75^NTR^-TRADD-mediated antiapoptotic pathway in breast cancer cell.

## Introduction

The death domain (DD) superfamily is one of the largest collection of structurally and functionally related protein interaction modules. DD-containing proteins play key roles in the activation of apoptotic, innate immunity and inflammatory signaling through the formation of oligomeric protein complexes with receptors, kinases and other proteins^1, 2^. DD-mediated signaling pathways are related to many important human diseases, hence a better understanding of their structure and function is of great biological importance. The DD superfamily comprises four subfamilies of domains with closer structural similarity, including the canonical DD, the death effector domain (DED), the caspase recruitment domain (CARD) and the pyrin domain (PYD)^3^. Members of the DD superfamily share a common structural fold, namely an isolated antiparallel helix bundle. Nevertheless, individual members exhibit different structural characteristics, including varied helix length and orientation as well as highly diverse electrostatic surfaces, a feature that is critical for the binding specificity of DDs^4, 5^.

DDs are present in a wide range of proteins, including several members of the tumor necrosis factor receptor (TNFR) superfamily and various intracellular signaling molecules, such as caspases and kinases^6^. The TNFR1-associated death domain protein (TRADD) is a multifunctional 34-kDa adaptor protein, consisting of two structurally distinct domains connected by a long linker peptide of 37 amino acid residues^7–10^. Its N-terminal domain folds into an α/β sandwich structure while its carboxyl-terminal domain is a DD (TRADD DD)^8, 9^. TRADD has several protein binding partners and participates in different signaling pathways, including NF-κB, apoptosis, necrosis and mitogen-activated protein (MAP) kinase activation^10, 11^. It binds to TNFR1 in a TNF dependent manner and serves as a platform to recruit additional proteins^7^. The N-terminal domain of TRADD interacts with the C-terminal domain of TNFR-associated factor 2 (TRAF2) and recruits TRAF2 to TNFR1 for activation of the NF-κB pathway^8, 12^. On the other hand, the TRADD DD interacts with other DD-containing proteins through homotypic DD-DD interactions. TRADD DD directly binds the DD of TNFR1^7^. It can also simultaneously bind to the DD of Fas-associated protein with death domain (FADD) to recruit FADD to TNFR1 for initiation of the apoptototic cascade^13^. It has been reported that TRADD can associate with the p75^NTR^ in (MCF-7) breast cancer cells upon receptor activation by neurotrophins (NT)^14^. The involvement of TRADD in p75^NTR^ signaling was shown to be required for NF-κB activation and control of antiapoptotic effects of neurotrophins in breast cancer cells. Upon TRADD depletion, p75^NTR^ was reported to induce cell death in conditionally immortalized striatal neurons^15^. These studies suggest that TRADD is crucial for balancing antiapoptotic and proapoptotic signaling pathways.

The structure of human TRADD DD has previously been determined by nuclear magnetic resonance (NMR) technique under an acidic condition of pH 4.2^9, 16^. However, its structure file is unavailable. At pH 4.2, TRADD DD adopt a characteristic DD fold, but the residues A199-S215 in its N terminus were completely disordered in solution^9^. Here, we present a different NMR solution structure of human TRADD DD in pure water, revealing a structure not previously seen in the DD superfamily and providing a better structural basis for studying TRADD DD-mediated signaling.

## Results and Discussion

### NMR Solution Structure Determination of TRADD DD

**Figure 1:**
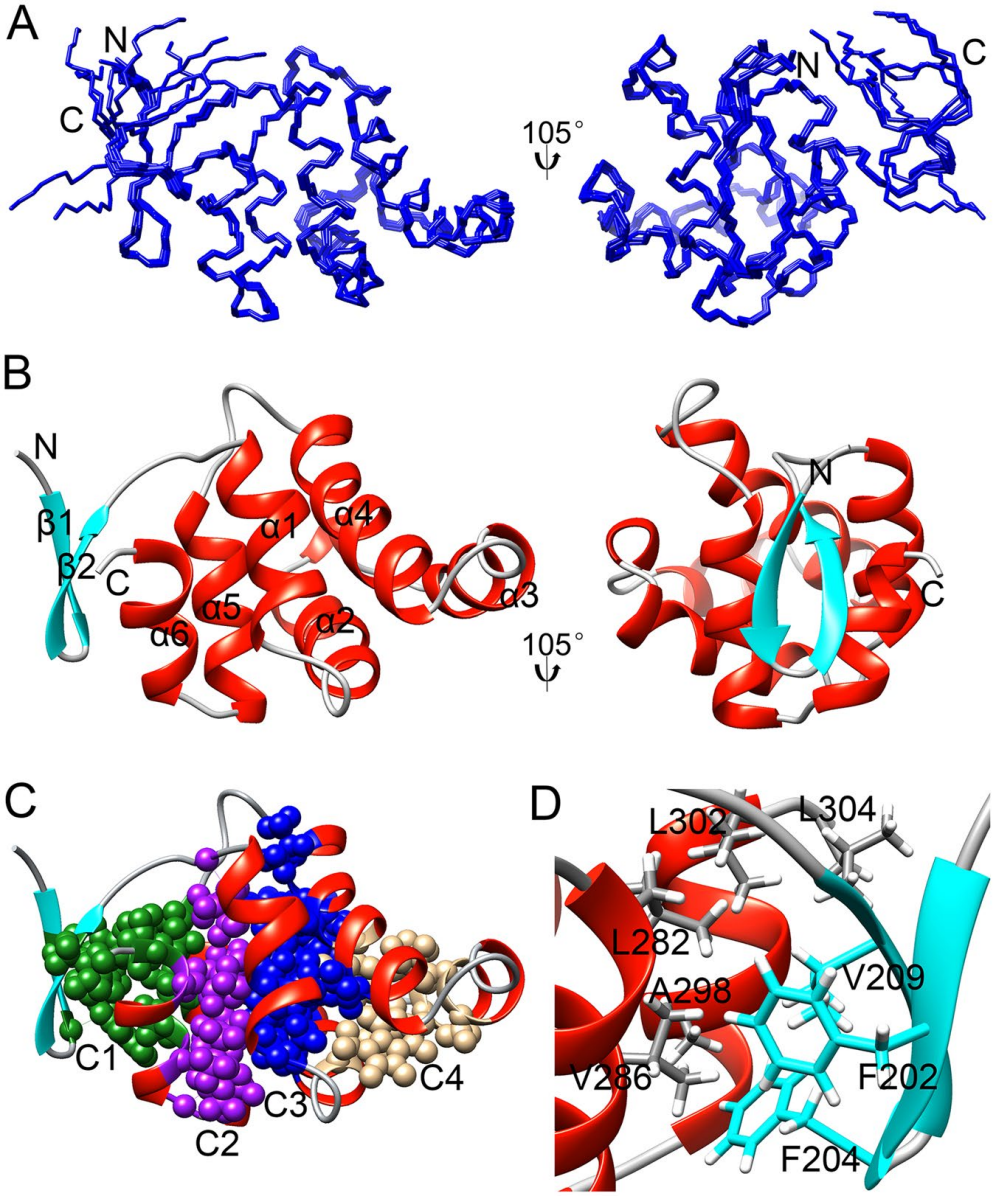
Solution structure of TRADD DD. (**A**) Superposition of the backbone heavy atoms of the ten lowest-energy structures of TRADD DD. (**B**) Ribbon drawing of the lowest-energy conformer of TRADD DD. (**C**) Four structurally distinct hydrophobic cores in TRADD. Core 1 (C1, green) is formed by a β-hairpin, helices α5 and α6; core 2 (C2, purple) and core 3 (C3, blue) are two central cores formed by three (α1, α5 and α6) and four (α1, α2, α4 and α5) helices, respectively; core 4 (C4, brown) is packed by helices α2, α3 and α4. (**D**) Hydrophobic core 1 involves 8 hydrophobic residues from one pair of β strands and one pair of α helices.

The structure of monomeric TRADD DD was determined by a total of 2990 nuclear Overhauser effect (NOE)-based distance restraints, including 547 long-range NOEs, which were obtained using uniformly ^15^N- and ^15^N/^13^C-labeled proteins with double and triple resonance NMR experiments. The structural statistics and root-mean-square deviation (RMSD) are in Table 1, and the superposition of the ensemble of 10 structures with lowest energy is shown in Fig. 1A. Although the C-terminal tail of residues T305-A312 is disordered, the globular domain of TRADD DD was defined with high precision. RMSD to the mean coordinate for residues T201-L304 is 0.22 ± 0.05 Å for the backbone atoms and 0.70 ± 0.05 Å for all atoms. This fine result can be attributed to ~28 NOE distance constraints per residue obtained in the 104-residue globular structure of TRADD DD (Table 1 and Fig. S1), for which nearly complete chemical shifts have been assigned. Ramachandran plot analysis of ordered regions placed 90.7% of residues in the “most favored regions” with 9.3% of residues in the “additional allowed regions” and no residue in the “generously allowed region” or “disallowed region”. Global quality Z scores were −1.28, 0.21, −0.12 and −1.38 for Verify3D, ProsaII(−ve), Procheck (phi-psi) and MolProbity Clashscore, respectively, indicating a good-quality structure of TRADD DD at the atomic level.

**Table 1:** NMR and refinement statistics TRADD DD.

**NMR distance & dihedral constraints**	
Distance constraints	
Total NOE	2990
Intra-residue	1171
Inter-residue	
Sequential (|i-j| = 1)	664
Medium-range (|i-j| ≤4)	608
Long-range (|i-j| ≥5)	547
Total dihedral angle straints^a^	180
**Structure Statistics**	
Violations (mean and s.d.)	
Distance constraints (Å)	0.30 ± 0.02
Dihedral angle constraints (°)	3.78 ± 0.24
Max. dihedral angle violation (°)	4.30
Max. distance constraint violation (Å)	0.37
Ramachandran Plot^b^	
Most favored regions	90.7%
Addtional allowed regions	9.3%
Generously allowed regions	0.0%
Disallowed regions	0.0%
Average RMSD (Å)^c^	
Heavy atoms	0.70 ± 0.05
Backbone atoms	0.22 ± 0.05

^a^Dihedral angle constraints were generated by TALOS+ based on Cα and Cβ chemical shifts.

^b^As determined by PROCHECK NMR in the ordered region of 201–304.

^c^Average r.m.s. deviation (RMSD) to the mean structure was calculated among 10 refined structures. Superimposed residues are 201–304. The total AMBER energy is −5175.88 ± 21 kcal/mol.

### Novel Structure of TRADD DD

**Figure 2:**
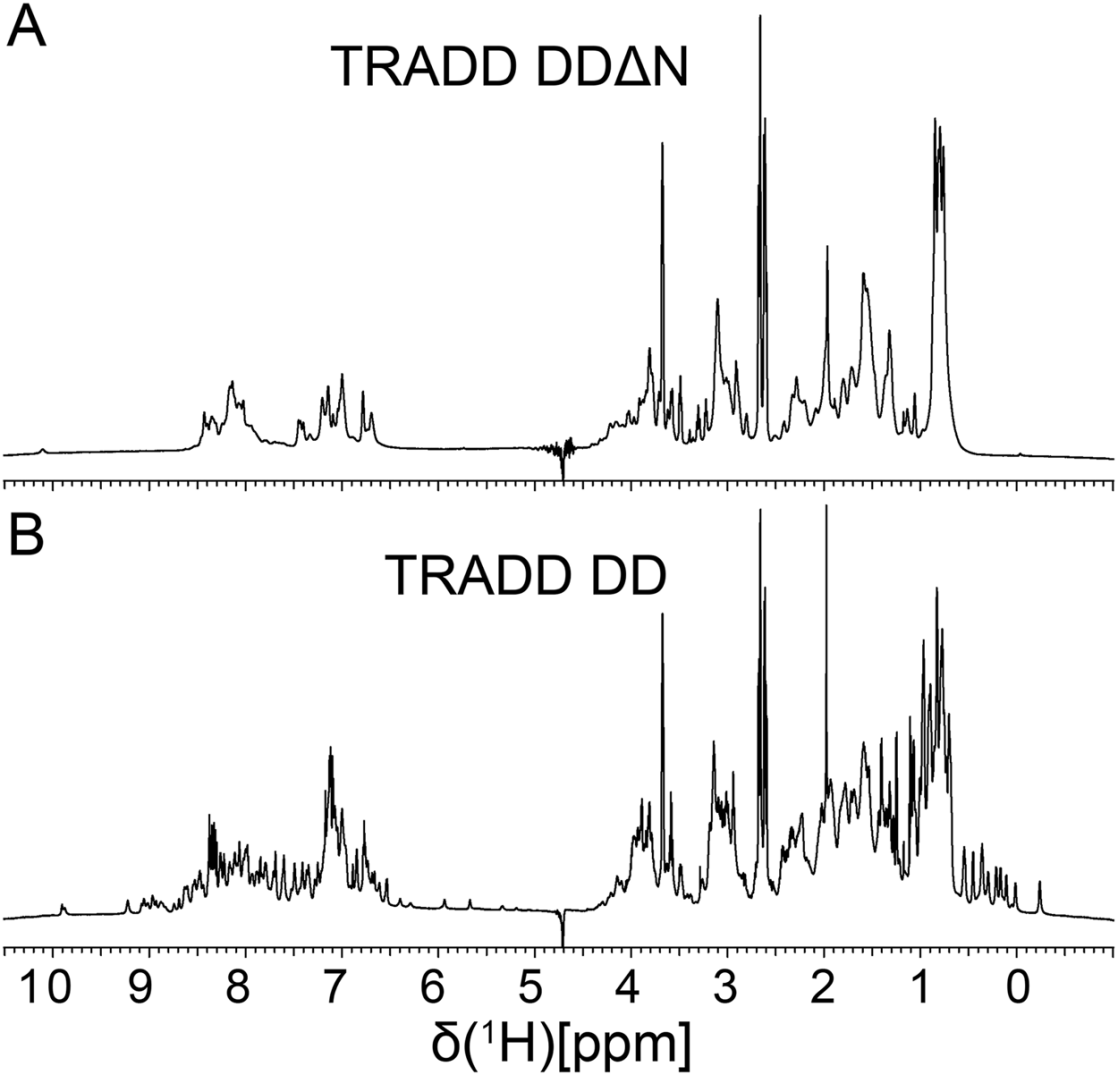
1D NMR spectra of TRADD DD. (**A**) 1D NMR spectrum of TRADD DDΔN (Δ199–214) indicating large aggregation of proteins in solution. (**B**) 1D NMR spectrum of full-length TRADD DD (199–312) showing a well-folded protein structure.

The solution structure of TRADD DD is composed of two antiparallel β-strands, six antiparallel α-helices and a short three-residue 3_10_-helix between helices α2 and α3 (Fig. 1B). Detailed inspection of the domain structure reveals that TRADD DD comprises two major super-secondary structures, a Greek key motif made of five α-helices (α1, α2, α3, α4 and α5) and a β-hairpin motif (β1 and β2) flanked by helices α5 and α6. The two motifs are tightly packed together to form four distinct hydrophobic cores (Fig. 1C). The packing of β1, β2, α5 and α6 forms the first bundle with a hydrophobic core (C1). A number of long-range and tertiary hydrophobic interactions from Leu, Val, Phe, and Ala are involved in the formation and stabilization of C1 (Fig. 1D and Fig. S2). Helix α1 together with α5 and α6 form a central bundle and a central hydrophobic core (C2). The other central bundle is made of two pairs of parallel helices (α1/α5 and α2/α4) that are packed approximately orthogonally to form the third hydrophobic core (C3). The last hydrophobic core (C4) is formed by three contiguous helices α2, α3 and α4. These four hydrophobic cores are in distinct regions of the domain as shown in Fig. 1C. Different faces of helices α1, α2, α4, α5 and α6 contribute to different hydrophobic cores. The peripheral strands β1/β2 and helix α3 contribute only to the packing of C1 and C4, respectively. The β-hairpin at the N-terminus of TRADD DD is critical for domain folding. Removal of this motif resulted in incorrect folding and aggregation of TRADD DD in water solution, as evident by very narrow amide- and CH_3_-proton dispersion in one-dimensional ^1^H NMR spectrum (Fig. 2).

A published structure of TRADD DD at pH 4.2 showed that the N-terminal segment of 17 amino acid residues exists in random-coil conformation, which is inconsistent with our high-resolution solution structure and N-terminal truncation study. A likely possibility is that this β hairpin motif undergoes structural unfolding or disassembling at low pH. Nevertheless, we cannot at present rule out the possibility of insufficient or incorrect NOE assignments in previous study.

### Structural comparison to other members of the DD superfamily

**Figure 3:**
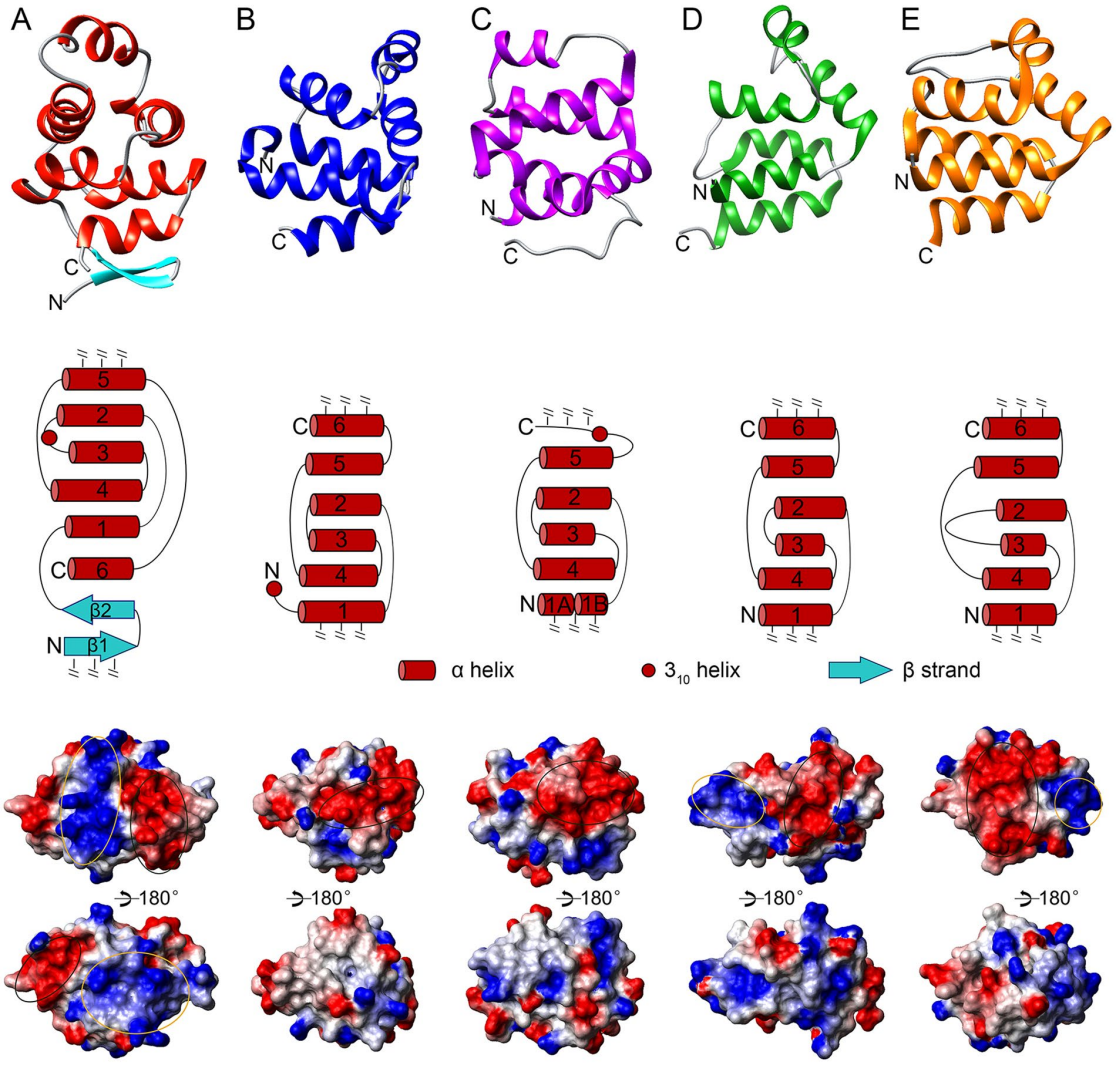
Structural comparison of TRADD DD and four different subfamilies of the DD superfamily. (**A**) TRADD DD. (**B**) p75^NTR^ DD (PDB ID: 2N97). (**C**) RIP2 CARD (PDB ID: 2N7Z). (**D**) FADD DED (PDB ID 2GF5). (**E**) ASC PYD (PDB ID: 1UCP). Upper panel: ribbon presentation; middle: 2D topology; lower: electrostatic surface. Disordered N-/C-terminal tails are not shown. Color code is blue for positive charges, red for negative charges, and white for neutral surface. Large positive and negative patches on the surfaces are circled in yellow and black, respectively.

A structural comparison between TRADD DD and four subfamilies of the DD superfamily shows expected similarities but also significant differences (Fig. 3). Most DDs have six α helices. The signature feature of canonical DDs is the folding of an all-helical Greek-key motif comprising the first five α helices, while the sixth helix is not involved in this motif^4^. In the case of the CARD domain, a kink angle usually exists in the middle of the first α helix which often breaks into two smaller ones. The sixth α helix of CARD domains may be replaced by a short 3_10_ helix, such as in the CARD of RIP2 (Fig. 3C). The all-helical Greek-key motif is highly conserved among four members of the DD superfamily, despite the absence of a helix α3 in a rare domain variant form from PYD subfamily^17^. A structure homology search conducted with the DALI server showed that the helical Greek-key motif of TRADD DD is structurally similar to canonical DDs with a highest Z score of ~10, indicating that TRADD DD can be classified into the DD superfamily (Fig. S3). A striking difference at the topology level is the presence of a β-hairpin motif between helices α5 and α6, which make it most unique in the DD superfamily. TRADD DD also exhibits significantly different surface characteristics when compared to p75^NTR^ DD, RIP2 CARD, FADD DED, and ASC (apoptosis-associated speck-like protein containing a caspase recruitment domain) PYD. Four highly positive and negative charge patches on the surface of TRADD DD suggest important roles for electrostatic interactions in TRADD DD-mediated signaling (Fig. 3A).

The DD superfamily has been intensively studied at cellular and structural levels due to its important role in mediating protein interactions underlying a wide range of signaling events. Alpha helices, including short 3_10_ helix, have until now been the only secondary structural element found in the DD superfamily. Therefore, the DD superfamily was believed to be comprised of all-helical globular domains. Our structural determination of TRADD DD by multi-dimensional NMR spectra unveils an α+β globular architecture of TRADD DD. In solution, TRADD DD behaves as an independently folded functional domain. Our results indicate that the β hairpin motif at the N-terminus is crucial for the structural integrity of the TRADD DD. The unusual combination of common β hairpin and all-helical Greek-key motifs was not previously seen in any DD. To the best of our knowledge, TRADD DD structure may represent a new class with both α and β structural elements in the DD superfamily. It is possible that other DDs for which we currently lack structural information also display a β motif, although secondary structure prediction fails to indicate any β structures for known DD sequences.

### Interaction between TRADD DD and other DDs

**Figure 4:**
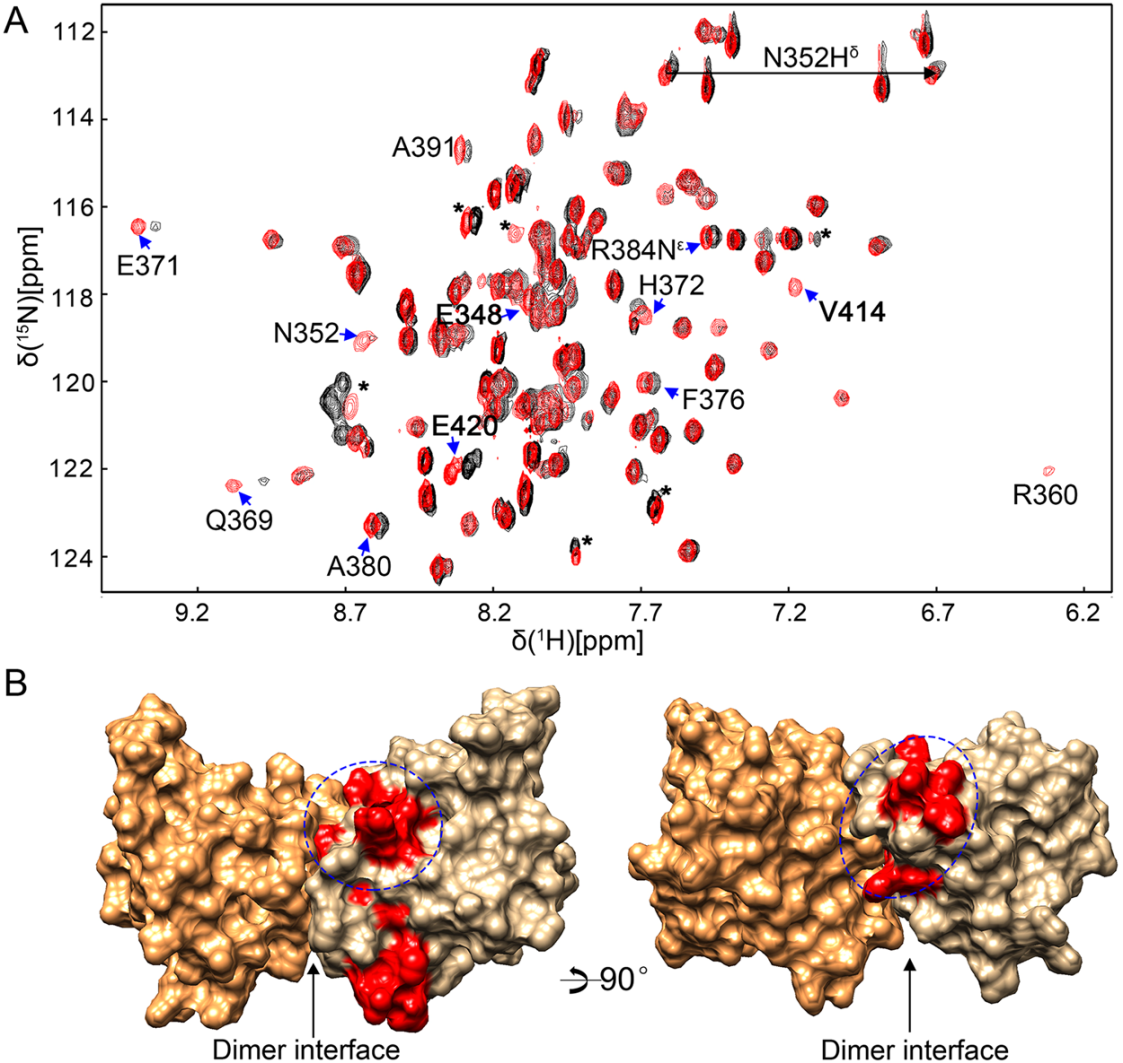
Interaction between TRADD DD and p75^NTR^ DD. (**A**) Expanded region of [^1^H-^15^N] HSQC spectra of p75^NTR^ DD in the absence (black) and presence (red) of TRADD DD at 28 °C in water. The concentration of TRADD DD is ~0.3 mM and the molar ratio of TRADD DD to p75^NTR^ DD is ~1:2. Perturbed residues are labelled. (**B**) Surface presentation of p75^NTR^ DD homodimer. p75^NTR^ DD homo-dimerization interface is indicated by an arrow. Most-perturbed residues on the structural region of p75^NTR^ DD are colored in red and circled in blue. *Cross peaks from the N-/C-terminal tails.

As a multifunctional signaling adaptor protein, TRADD DD has been reported to interact with multiple death receptors, including p75^NTR^, in different cellular contexts^11^. In breast cancer cells, the TRADD DD was reported to bind p75^NTR^ for the activation of NF-κB, similar to the signaling mediated by TNFR1. Based on TRADD truncation studies, the DD of TRADD was found to be responsible for binding. The p75^NTR^ DD has been proposed to initiate the NF-κB-mediated survival pathway via interaction with the TRADD DD. We examined the interaction of TRADD and p75^NTR^ DDs by performing NMR titration studies at different TRADD DD concentrations in the presence of dithiothreitol (to avoid the formation of disulfide-bonded dimers) and in the absence of phosphate ions (to prevent non-covalent homodimerization of p75^NTR^ DD) as described earlier^18^. Upon addition of unlabeled TRADD DD, ^15^N-labelled p75^NTR^ DD monomer showed small chemical shift or intensity changes in the 2D ^1^H-^15^N HSQC spectrum, which is a characteristic of low affinity binding in µM-mM range. More than 10 residues that could be involved in binding TRADD DD were identified in the DD of p75^NTR^ (Fig. 4A). Mapping of most-perturbed residues on the surface of p75^NTR^ DD showed that they are very close to the p75^NTR^ DD homodimerization interface (Fig. 4B). This finding suggests that recruitment of TRADD to p75^NTR^ may require the separation of the p75^NTR^ DD homodimer, similar to our previous observations with the CARD of RIP2^18^. Nevertheless, their binding sites on p75^NTR^ DD could be completely different.

In this study, we observed for the first time a direct homotypic interaction between TRADD and p75^NTR^ DDs. The small degree of chemical shift perturbation also indicates that the DDs do not undergo global conformational changes upon complex formation. However, dissociation of p75^NTR^ DD homodimer could be a prerequisite for the binding of TRADD DD to p75^NTR^ DD since this binding is dependent on the stimulation of NGF which has been showed to trigger the separation of p75^NTR^ DD homodimer. A better understanding of the mechanism underlying NGF-induced recruitment of TRADD by p75^NTR^ will require the structural determination of the complex between TRADD DD and p75^NTR^ DD.

TRADD DD also interacts with FADD DD and TNFR1 DD. An alanine-scanning mutagenesis study of the TRADD DD identified some structural determinants and dissected TRADD DD-mediated signaling^19^. However, discrete surfaces on the TRADD DD responsible for different activities could not be identified due to the lack of consideration for the structural integrity of TRADD DD. The strategy of multiple contiguous mutations in individual constructs used in the alanine-scanning mutagenesis study could result in local or global structure disruption and the failure of epitope identification. Single selective mutation was also introduced to study how TRADD DD interacts with FADD DD and TNFR1 DD^20^. The putative FADD DD and TNFR1 DD binding sites on TRADD DD were found to partially overlap, which seems to contradict with previous biochemical studies showing co-immunoprecipitation of trimeric TNFR1:TRADD:FADD complex (Fig. S4)^7^. Based on our solution structure, however, it’s possible that 11 putative TNFR1 DD binding residues on TRADD DD could make up two non-overlapping binding sites. FADD DD binding site on TRADD DD could only overlap with one of them, leaving the other for binding TNFR1 DD.

In summary, our solution structure of TRADD DD determined by multi-dimensional NMR spectroscopy uncovered a novel β-hairpin structural element in the DD structure. It may represent a new class in the DD superfamily and provide a structural basis for studying interactions between TRADD DD and other DD-containing proteins.

## Methods

### Cloning of TRADD DD

The cDNA of human TRADD DD (199-312) was amplified from total human embryonic stem cell cDNA and subcloned into a pET32-derived expression vector between BamH I and EcoR I restriction sites. Construct integrity was confirmed by DNA sequencing. The recombinant TRADD DD contains 13 additional residues (MHHHHHHSSGRGS) at the N-terminal end, including a hexa-His tag.

### Protein Purification and NMR Sample Preparation

Unlabeled TRADD DD and TRADD DDΔN were over-expressed in *E. coli* strain SoluBL21 (DE3) in LB or M9 minimal medium. Protein samples were purified using Ni-NTA affinity chromatography followed by size-exclusion chromatography (Superdex 75). Isotopic labeling was carried out by expressing the proteins in M9 minimal medium containing ^15^N-NH_4_Cl and ^13^C-labeled glucose as the sole sources of nitrogen and carbon. NMR sample contained ~0.6 mM ^13^C,^15^N-labeled TRADD DD in water and 5% D_2_O. Since TRADD DD contains one Cys residue, 10 mM D_10_-DTT was also included in the NMR samples to avoid intermolecular disulfide bond formation.

### NMR Spectroscopy Experiments and Structure Determination

All NMR experiments were performed on a Bruker AVANCE 800 MHz NMR spectrometer with a cryogenic probe at 28 °C. NMR spectra were processed with NMRPipe^21^ and analyzed with NMRDraw and NMRView supported by a NOE assignment plugin^22^. Resonance assignments of backbone, aliphatic, and aromatic side chains were obtained using previously described methods^23, 24^. The chemical shift values of backbone C_α_, C_β_ and H_N_ were analyzed by TALOS+ to predict backbone torsion angles^25^. Intramolecular NOE restraints were obtained from 4D time-shared ^13^C, ^15^N-edited NOESY spectra^26^. Ambiguous NOEs were assigned with iterated structure calculations by DYANA^27^. Based on peak volume, NOE values were binned into short (1.8–2.8 Å), medium (1.8–3.4 Å) and long (1.8–5.5 Å) distances. Final structure calculation was started from 100 conformers. 10 conformers with the lowest final target function values were selected for energy minimization in AMBER force field^28^. The mean structure was obtained from the 10 energy-minimized conformers. PROCHECK-NMR^29^ was used to assess the quality of the structures. Protein Structure Validation Suite (PSVS)^30^ was applied to assess the overall structure quality. Structure homology search was performed in DALI server^31^. All the structural figures were made using UCSF Chimera^32^ or MOLMOL^33^.

### Accession numbers

Assignments have been deposited in the Biological Magnetic Resonance Data Bank (BMRB: 36084). The structure of TRADD DD has been deposited in the PDB (PDB: 5XME).

## Electronic supplementary material

Supplementary information
