# Supplementary material for: Structure of the C-terminal domain of TRADD reveals a novel fold in the death domain superfamily
Source: Sci Rep. 2017 Aug 1;7:7073. doi: 10.1038/s41598-017-07348-9 (PMC5539145; doi:10.1038/s41598-017-07348-9)

Supplementary Information

**Structure of the C-terminal domain of TRADD reveals a novel fold in the death domain superfamily**

Ning Zhang^1^, Wensu Yuan^1^, Jing-Song Fan^2^, Zhi Lin^1,3,4^

^1^School of Life Sciences, Tianjin University, Tianjin, 300072, P.R. China

^2^Department of Biological Sciences, National University of Singapore, Singapore, 117543, Singapore

^3^Department of Physiology, National University of Singapore, Singapore, 117593, Singapore

^4^Life Sciences Institute, National University of Singapore, Singapore, 117456, Singapore

Correspondence and requests for materials should be addressed to Z.L. (email: linzhi@linzhi.net)

**Figure S1. NOE statistics for each amino acid resides of TRADD DD**


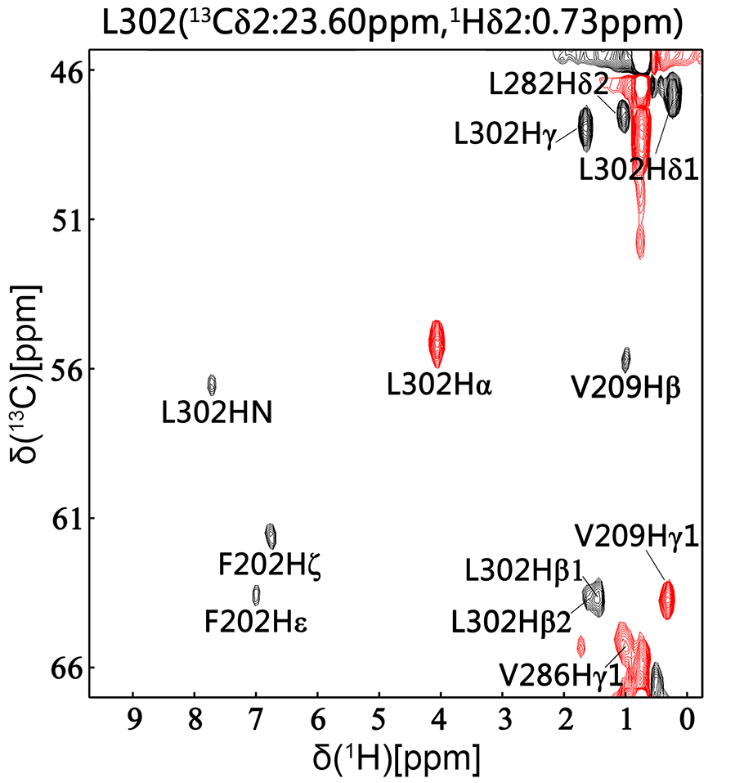

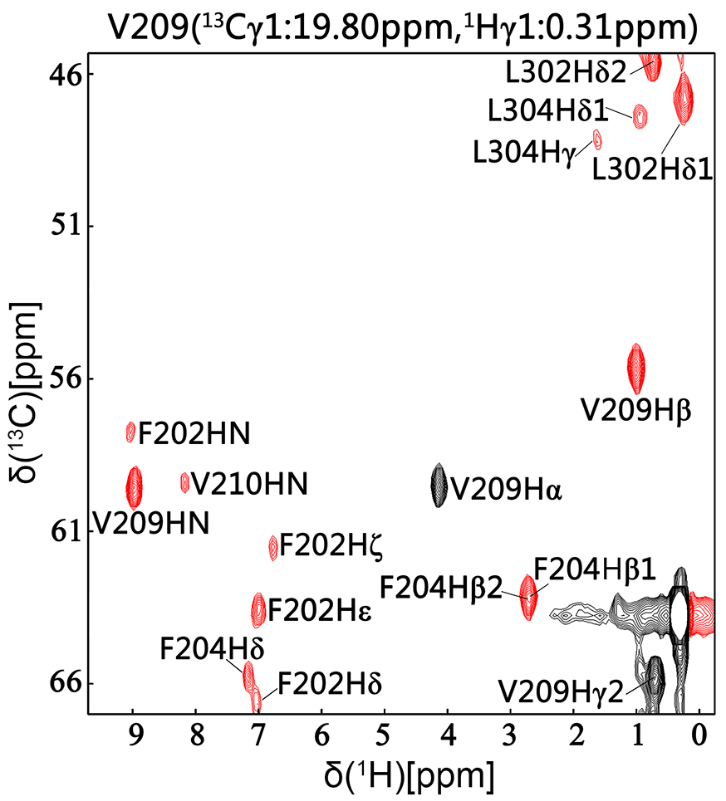


**Figure S2****. Representative slices from 4D NOESY spectra recorded on TRADD DD.** (**A**) 4D ^13^C, ^13^C-edited NOESY. (**B**) 4D ^13^C, ^13^C-edited NOESY. Each F_1_(^1^H)-F_2_(^13^C) slice is labeled with proton identities. F_3_(^13^C) and F_4_(^1^H) frequencies in ppm with assigned residues are indicated at the top of each slice.

A

B


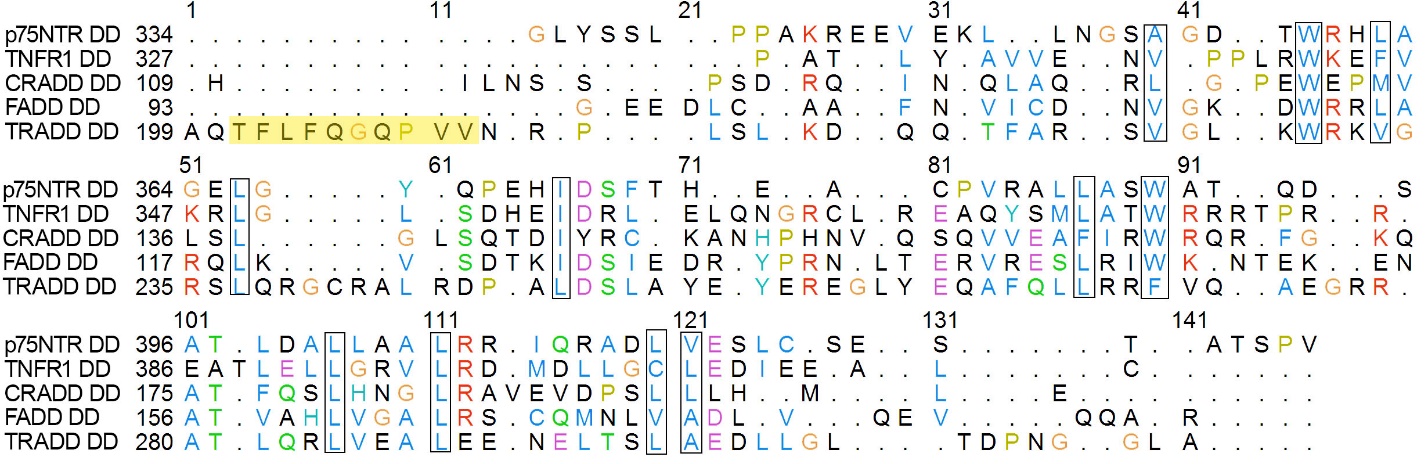

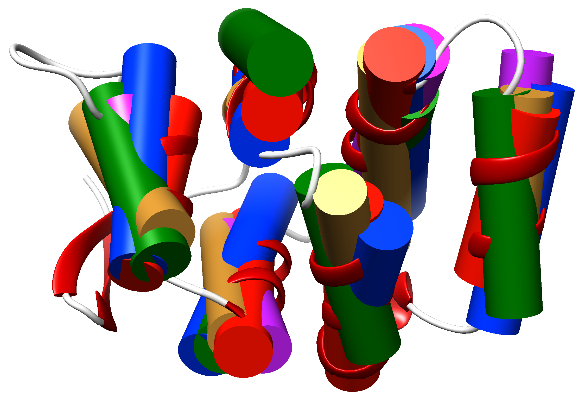

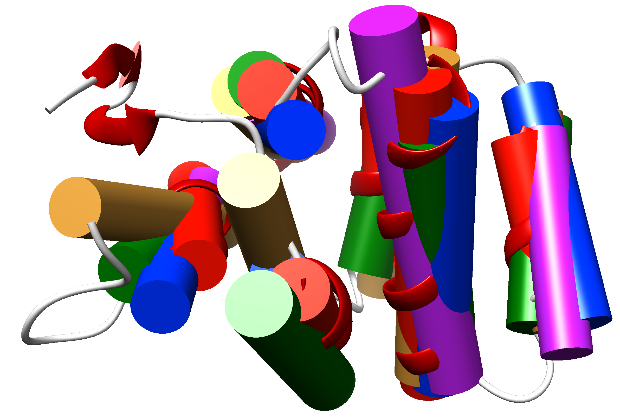

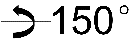


B

A

**Figure S3. Structure-based sequence alignment among DDs.** (**A**). Multiple structure-based sequence alignment among p75^NTR^ DD, TNFR1 DD, CRADD DD, FADD DD and TRADD DD. The conserved hydrophobic core resides are boxed and the resides involved in β-hairpin formation in TRADD DD are highlighted in yellow. Sequence identity between TRADD DD and other DDs ranges from 16%-24%. (**B**) TRADD DD (red) is superimposed with p75^NTR^ DD (green), TNFR1 DD (blue), CRADD DD (magenta) and FADD DD (orange). α helices are shown as columns.

α1

α2

α3

α4

α5

α6

**Figure S4. Putative binding sites for TNFR1 DD and FADD DD.** Surface presentation of TRADD DD. N- and C-termini are indicated. Residues that affected interactions with TNFR1 DD (11 residues) and FADD DD (6 residues) are colored in green (**A**) and blue (**B**), respectively. Overlapping residues for binding TNFR1 DD and FADD DD are labelled in **A**. 11 residues on TRADD DD for binding TNFR1 DD could form two non-overlapping binding sites, which are circled in red.


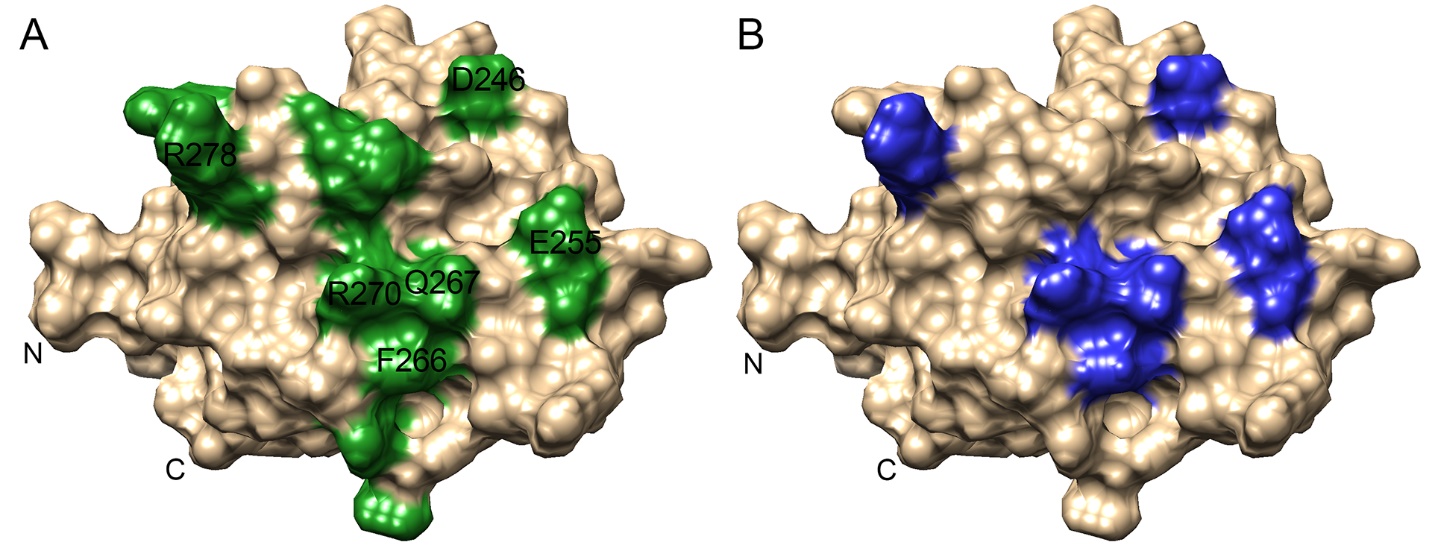

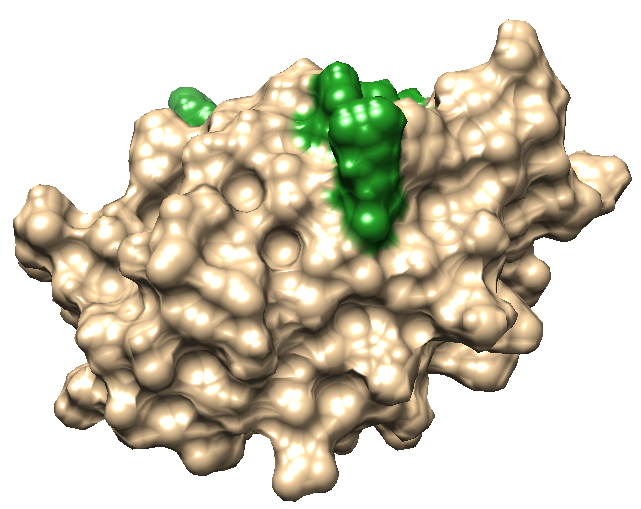

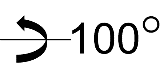

Supplement: Supplementary file 1 — Supplementary information [file 41598_2017_7348_MOESM1_ESM.docx]
